# Deep learning-based radiomics and pathomics for decoding tumor microenvironment and predicting immunotherapy outcomes in gastric cancer

**DOI:** 10.3389/fimmu.2026.1970256

**Published:** 2026-09-18

**Authors:** Xinyi Shen, Zeyu Liu, Wenjuan Yu, Fei Li, Yuxiao Lu, Ming Li, Ping Sun, Aina Liu

**Affiliations:** 1 Department of Oncology, The Affiliated Yantai Yuhuangding Hospital of Qingdao University, Yantai, China; 2 Department of Pathology, The Affiliated Hospital of Qingdao University, Qingdao, China

**Keywords:** deep learning, gastric cancer, immunotherapy, pathomics, radiomics, tumor microenvironment

## Abstract

Although immune checkpoint inhibitors have reshaped the therapeutic landscape of gastric cancer, durable responses are achieved in only a minority of patients. The tumor microenvironment, particularly immune-cell composition and spatial organization, critically modulates cancer progression and the efficacy of immune checkpoint inhibitors. Nevertheless, the lack of clinically accessible approaches for spatially resolving the tumor immune contexture, whether by direct pathological quantification or indirect radiomic inference, has hindered a mechanistic understanding of how it dictates immunotherapy outcomes. Deep learning-based radiomics and pathomics extract high-dimensional phenotypic information from radiological and pathological images, thereby characterizing tumors at macroscopic and microscopic scales. Each modality, however, captures only a partial view of the tumor microenvironment. Multimodal models can integrate complementary imaging features associated with tumor heterogeneity and spatial immune organization, thus improving predictive performance and linking multimodal features to immune-related biological processes. In this review, we summarize recent advances in deep learning-based radiomics, pathomics, and multimodal frameworks for decoding the immune microenvironment and predicting immunotherapy outcomes in gastric cancer. We also discuss their potential to complement established biomarkers and support personalized treatment decisions. Finally, we highlight key challenges, including limited data availability, interinstitutional variability, insufficient external validation, poor interpretability, and barriers to clinical implementation. Addressing these limitations may accelerate the development of robust imaging biomarkers for guiding immunotherapy decisions in gastric cancer.

## 1 Introduction

Gastric cancer (GC) is one of the most common malignancies and a leading cause of cancer-related death worldwide (1). Owing to its often asymptomatic or nonspecific clinical presentation in early-stage disease, insufficient coverage of screening programs, and unequal distribution of healthcare resources across different regions, a substantial proportion of patients are diagnosed with locally advanced or metastatic disease at initial presentation (2, 3). Although continuous advances in conventional therapeutic strategies, including surgical resection, perioperative chemotherapy, and chemoradiotherapy, have improved clinical outcomes to some extent, the overall prognosis of patients with advanced GC still remains poor (3). Currently, the median overall survival of patients with advanced GC receiving systemic therapy is only approximately 13–20 months (4–6).

In recent years, immunotherapy, particularly immune checkpoint inhibitors (ICIs) targeting the programmed cell death protein 1/programmed death-ligand 1 (PD-1/PD-L1) axis, has redefined the first-line treatment landscape for advanced GC. By relieving inhibitory signals that impair T-cell function and restoring antitumor immune responses, ICIs have provided durable clinical benefits for a subset of patients with advanced disease (7–9). Several phase III trials, including CheckMate-649 (4) and KEYNOTE-859 (5), have demonstrated that ICIs combined with chemotherapy significantly prolong progression-free survival (PFS) and overall survival (OS) in patients with locally advanced or metastatic GC. Accordingly, the US Food and Drug Administration has approved nivolumab and pembrolizumab as a first-line treatment for advanced GC. Although ICIs have become an integral component of advanced GC treatment, only a proportion of patients achieve clinically meaningful and durable responses. Therefore, improving therapeutic benefit, accurately identifying patients who are most likely to respond, and minimizing ineffective treatment and associated toxicities remain central to the development of precision immunotherapy for GC.

At present, the prediction of immunotherapy efficacy in GC relies primarily on a range of molecular and immune-related biomarkers, including the PD-L1 combined positive score (CPS), microsatellite instability (MSI) (10), tumor mutational burden (TMB) (11), and Epstein-Barr virus (EBV) (12). However, the clinical utility of these biomarkers remains constrained by several factors, including the lack of fully standardized testing criteria (13–15), relatively high costs (16), and limited applicability across patient populations (12, 17). More importantly, most established biomarkers depend on localized tissue biopsies and capture only individual biological dimensions (18), such as tumor immunogenicity (19), immune checkpoint activation (20–22), or genomic instability (23). This limits their ability to dissect the complexity and spatially heterogeneous tumor microenvironment (TME), which crucially influences cancer progression and therapeutic outcomes (19, 24). Accumulating evidence suggests that the abundance and spatial organization of immune cells across intratumoral and peritumoral regions are associated with ICI response, as distinct immune phenotypes of the TME show markedly different treatment sensitivities (25, 26). Radiomics and pathomics provide complementary tumor characterization at macroscopic and microscopic scales by capturing imaging phenotypes and tissue-level cellular morphology and spatial organization, respectively (27, 28). Therefore, biomarkers integrating both compartments may better characterize TME immune phenotypes and support precise clinical decision-making (29, 30).

Deep learning can automatically extract high-dimensional semantic features associated with tumor heterogeneity from medical images such as computed tomography (CT), magnetic resonance imaging (MRI), and whole-slide images (WSIs), while facilitating the direct quantification of immune-cell infiltration in pathology images and its indirect inference from radiological phenotypes. These features enable the development of predictors that link imaging and pathological phenotypes to TME states and, ultimately, to immunotherapy response and prognosis (31). Its end-to-end learning paradigm reduces reliance on handcrafted features and identifies subtle visual and spatial patterns that may be overlooked by conventional assessment (32).

Existing studies have combined deep learning with radiomics and pathomics to predict tumor node metastasis (TNM) stage and response to neoadjuvant chemotherapy in GC, with encouraging preliminary performance. These advances pave the way toward a clinically actionable imaging biomarker that not only predicts prognosis or treatment responses but also provides deeper insights into the immune landscape of the TME and addresses the key clinical questions in GC immunotherapy: which patients are most likely to benefit from ICIs, and which are most likely to exhibit primary resistance. In this review, we summarize advances in deep learning-based radiomics, pathomics, and multimodal models for characterizing TME function, improving existing biomarkers, and predicting responses to immunotherapy in GC. We further discuss current challenges in model interpretability and clinical translation, with the aim of supporting the integration of imaging biomarkers into precision immunotherapy for GC. The overall framework of this review is summarized in **Figure 1**. Relevant publications were identified through targeted searches of PubMed and Web of Science up to August 2026, using combinations of terms related to gastric cancer, immunotherapy, tumor microenvironment, deep learning, radiomics, pathomics, and multimodal learning. Studies were included if they evaluated deep learning-based radiomics, pathomics, or multimodal imaging approaches in GC in relation to the TME, immunotherapy-related biomarkers, treatment response, or clinical outcomes. Studies were excluded if they lacked direct relevance to the scope of this review, did not involve an imaging, radiomics/pathomics, or deep-learning component, provided insufficient methodological or outcome information, or represented duplicate or superseded reports. Reference lists of relevant articles were additionally screened to identify eligible studies.

**Figure 1:**
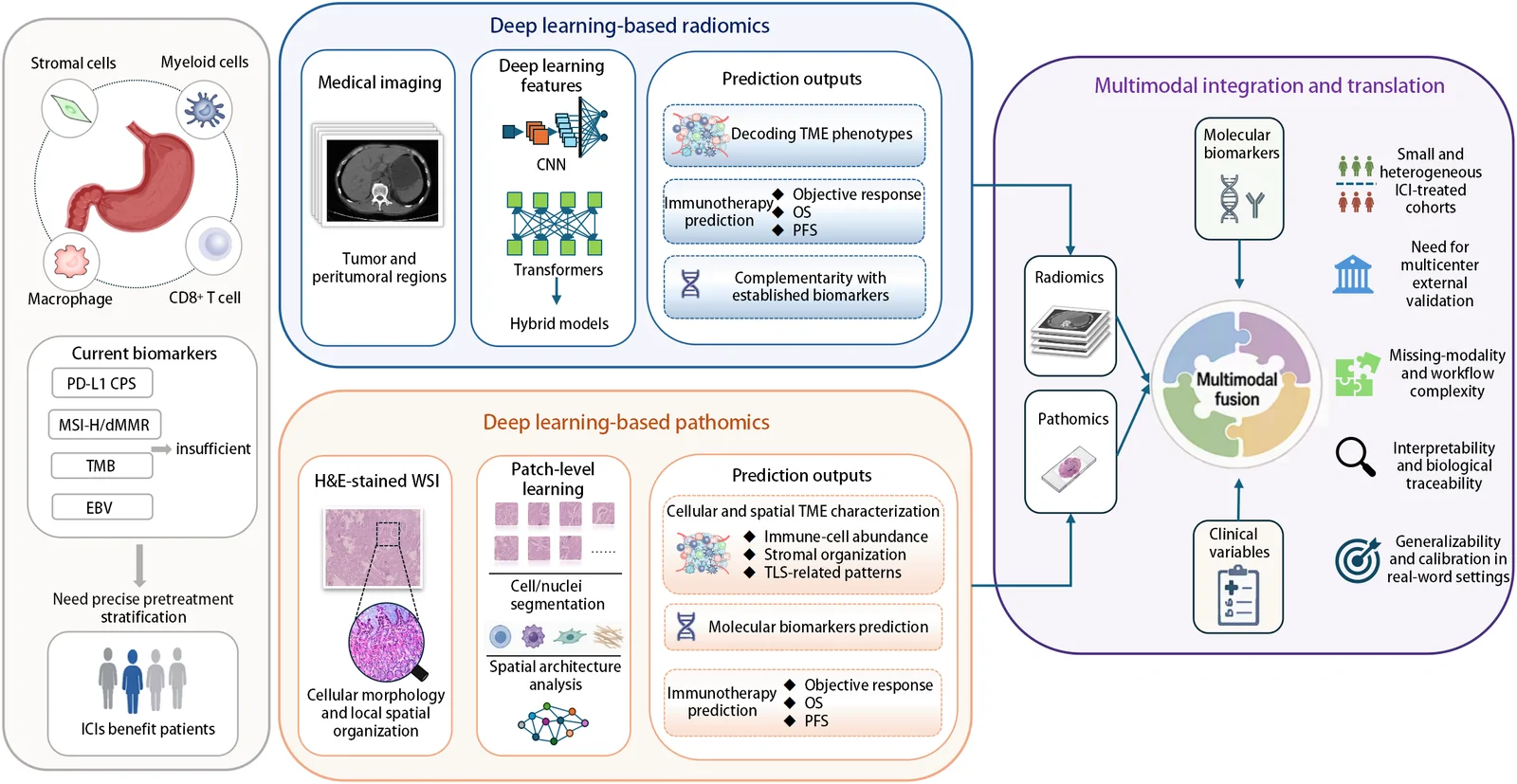
Deep learning-based radiomics and pathomics for decoding the tumor microenvironment and predicting immunotherapy outcomes in gastric cancer. ICIs benefit only a subset of patients with GC, and established biomarkers, including PD-L1 CPS, MSI-H/dMMR, TMB, and EBV status, remain insufficient. Deep learning radiomics uses CNNs, Transformers, and hybrid architectures to extract whole-tumor and peritumoral CT features as macroscopic surrogates of immune phenotypes for predicting ICI response, PFS, and OS. Pathomics applies patch-level learning, cell segmentation, and spatial architecture analysis to H&E-stained WSIs to characterize immune-cell abundance, stromal organization, and TLS-related patterns. Multimodal models integrate radiomic and pathomic features with molecular biomarkers and clinical variables, linking macroscopic imaging phenotypes with microscopic tissue characteristics. Clinical translation remains limited by poor interpretability, small heterogeneous cohorts, missing modalities, and insufficient external validation. Therefore, future studies should prioritize prospective multicenter validation, longitudinal monitoring, modality-flexible models, and biologically anchored biomarkers. CNN, convolutional neural network; CPS, combined positive score; CT, computed tomography; dMMR, deficient mismatch repair; EBV, Epstein-Barr virus; GC, gastric cancer; H&E, hematoxylin and eosin; ICI, immune checkpoint inhibitor; MSI-H, microsatellite instability-high; OS, overall survival; PD-L1, programmed death-ligand 1; PFS, progression-free survival; TMB, tumor mutational burden; TME, tumor microenvironment; TLS, tertiary lymphoid structure; WSI, whole-slide image.

## 2 Applications of deep learning-based radiomics in GC immunotherapy

The efficacy of ICIs is influenced by the cellular composition and spatial organization of the TME, including effector T cells, immunosuppressive myeloid cells, stromal barriers, and vasculature (33–35). Because biopsy-based assessment samples only a limited tumor region, deep learning-based radiomics can automatically capture imaging-derived surrogate features associated with TME-related phenotypes across the whole tumor and peritumoral regions, potentially reducing spatial sampling bias (36, 37). These imaging-TME associations may reflect macroscopic changes in tumor density and texture driven by immune infiltration, stromal remodeling, and angiogenesis in routine medical images (38, 39).

Notably, the presence of imaging associations cannot directly identify immune-cell lineages, functional states, or signaling activity. Imaging features can only be interpreted as TME-related surrogates, whose biological meaning requires validation with histopathological, immunohistochemical, or transcriptomic reference data. Given these limitations, deep learning-based radiomics has been investigated for the characterization of the TME, prediction of clinical outcomes in ICI-treated patients, and integration with established biomarkers.

### 2.1 Imaging-based TME decoding and biology-guided multitask learning

Immune-inflamed, immune-excluded, and immune-desert phenotypes are commonly distinguished by effector T-cell abundance and spatial distribution relative to tumor parenchyma and stroma (40, 41). These immune phenotypes have been demonstrated to show distinct sensitivities to ICIs (42). Although these microscopic immune-cell distributions cannot be directly resolved on routine CT, the associated stromal and vascular changes may manifest as quantifiable imaging phenotypes (43).

In GC, Chen et al. (44) reported that CT radiomics-defined risk groups exhibited distinct immune profiles, with low-risk tumors showing stronger CD8+ T-cell signatures and greater enrichment of TNFα/NF-κB, IL-2/STAT5, and IL-6/JAK/STAT3 signaling than high-risk tumors. These associations suggest that radiomic stratification may reflect immune-related differences in the TME and may further help identify TME phenotypes potentially relevant to immunotherapy response (45).

Biology-guided multitask learning (MTL) provides a more explicit strategy than association-based radiomics for linking imaging features to the TME. Instead of training a model only against a clinical endpoint, TME-related measurements can be incorporated as auxiliary labels that regularize the learned feature representation (46–48). Given that CD3 and CD8 reflect lymphocyte infiltration (49) and serve as the basis for ImmunoScore (IS), and that periostin (POSTN) marks activated fibrotic stroma (50–53), Jiang et al. (54) developed a MTL convolutional neural network (CNN) to classify four TME states, defined by high or low IS and POSTN expression, while jointly predicting disease-free survival (DFS). When the model was subsequently evaluated in an anti-PD-1 cohort, the CT-predicted TME classes stratified objective response and PFS. Similarly, Sang et al. (55) developed attention-enhanced residual Swin Transformer (AER-SwinT) and further validated that imaging phenotypes characterized by high IS and low POSTN were associated with higher response rates and longer PFS in two anti-PD-1 cohorts. Moreover, the TME labels were derived from tissue measurements, and CT-based predictions remain indirect because CT imaging does not directly visualize immune cells, cancer-associated fibroblasts, or their spatial interactions within the TME (41, 56). Although these MTL models collectively provide a biological bridge between CT phenotypes and treatment outcomes, their development requires immunohistochemistry (IHC)-derived labels, which limits their clinical translational potential.

### 2.2 Deep learning-based radiomics for predicting immunotherapy response and clinical outcomes in gastric cancer

Based on these TME associations, deep learning-based radiomics has been investigated for its ability to predict clinical outcomes in patients with GC receiving immunotherapy. These endpoints capture distinct clinical dimensions, namely objective response reflecting tumor shrinkage over a defined assessment period, and PFS/OS capturing the time to progression and time to death, respectively.

For response classification, Jiang et al. (54) reported that their TME classification framework discriminated responders from nonresponders in an anti-PD-1 cohort. Building on this line of research, Sang et al. (55) modified the model architecture to extend the model evaluation beyond response classification to prognostic stratification. The resulting model not only discriminated responders from nonresponders in two independent anti-PD-1-treated cohorts, with area under the curve (AUC) values of 0.735 (95% confidence interval [CI]: 0.665–0.804) and 0.697 (95% CI: 0.562–0.832), respectively, but also significantly stratified PFS across the predicted TME classes (p < 0.001). Shifting the focus from treatment response to survival prediction, Gou et al. (57) developed an ensemble CNN model to predict 1-year survival in patients with GC receiving immunotherapy, and its risk stratification was also associated with OS.

Model architecture determines how imaging information is represented. CNNs, particularly ResNet- and Visual Geometry Group (VGG)-based networks, can automatically learn local texture and spatial patterns directly from images that may not be adequately described by handcrafted features (58, 59). Gou et al. (57) exemplified this approach by constructing an ensemble of one VGG network and four ResNet networks for GC survival stratification. By integrating representations learned by multiple CNN architectures, this approach avoided reliance on predefined radiomic features and reduced dependence on any single network. Furthermore, Transformers use self-attention to model broader contextual relationships (60–63), whereas hybrid CNN-Transformer architectures attempt to combine local and long-range information (60–62). Yuan et al. (64) combined CNN with a Swin Transformer to model both global context and voxel-level relationships, achieving strong performance across multicenter datasets. Moreover, Sang et al. (55) integrated residual CNN and Swin Transformer components to derive a CT-based TME score. These models typically use CNN modules to extract local texture and morphological details, while self-attention or Transformer modules capture long-range dependencies and global contextual information, thereby enabling a more comprehensive analysis of the pronounced heterogeneity and diverse imaging phenotypes of GC (65, 66).

Overall, no architecture is uniformly superior. CNNs may be advantageous in small datasets because of their stronger spatial inductive biases compared with Transformers (58), whereas Transformers can model broader contextual relationships when adequate data or pretraining is available (67). Hybrid or hierarchical models can balance these properties, but increased complexity may lead to overfitting, unstable calibration, and limited reproducibility. Model selection should therefore consider cohort size, image protocol, segmentation quality, pretraining, endpoint definition, and independent external validation rather than architecture alone (63).

### 2.3 Comparison and complementarity between deep learning radiomics and established biomarkers

Established biomarkers capture isolated dimensions of tumor immunity but incompletely represent spatial and temporal TME heterogeneity. CPS, for example, is based on localized tissue sampling and may be affected by interobserver variability and limited reproducibility (44, 68). In contrast, deep learning-based radiomics offers non-invasive and longitudinal assessment of heterogeneity across the tumor and peritumoral regions, thereby complementing information obtained from localized tissue biopsy.

For discriminating objective response, Jiang et al. (54) reported that the imaging-predicted TME classes achieved a higher AUC (0.753, 95% CI: 0.692–0.814) than CPS (0.646, 95% CI: 0.580–0.713). Combining both in a decision-tree model increased the AUC to 0.806 and further separated responses within CPS categories. Consistently, Sang et al. (55) reported that combining the imaging-derived TME score with CPS yielded AUCs of 0.782–0.828 across two cohorts (p < 0.05). Although clinical utility has not been established, these findings suggest incremental value beyond PD-L1 CPS expression within the study datasets. Unlike PD-L1 CPS, direct quantitative comparisons between deep learning-based radiomics and molecular biomarkers such as MSI/mismatch repair (MMR), EBV, or TMB for the same immunotherapy outcome are still limited in GC. This may partly reflect the low prevalence of molecularly defined subgroups such as MSI-high (MSI-H), which means that many radiomics immunotherapy cohorts contain too few MSI-H cases to support reliable direct comparison with imaging models, whereas CPS is available across a much larger proportion of patients and therefore more readily serves as a direct comparator. In addition, radiomics may refine molecular subgroups linked to immunotherapy sensitivity rather than directly compete with established molecular biomarkers. GC with MSI-H/deficient mismatch repair (dMMR) generally shows higher sensitivity to immunotherapy.

Nevertheless, primary resistance occurs in a proportion of patients (69–71), highlighting substantial heterogeneity within this subgroup. Jiang et al. (72) constructed a deep learning-based CT model and found that radiomics-derived stratification within MSI-H tumors was associated with distinct immune-infiltration patterns, suggesting that biological heterogeneity exists within MSI-H GC. Nevertheless, a checkpoint blockade cohort was not included, nor was immunotherapy response assessed. Future studies should determine whether this imaging-derived MSI-H/dMMR phenotype predicts objective response and survival in patients receiving ICIs and provides incremental value beyond tissue-based MSI/MMR testing.

EBV-positive GC similarly exhibits frequent lymphocyte infiltration and PD-L1 upregulation (73, 74). Nevertheless, EBV status has not been established as a treatment-specific biomarker (75), and a CT radiomics model that predicts EBV status may not directly indicate ICI response. More broadly, imaging-based inference of MSI or EBV is best used to prioritize confirmatory testing rather than replace validated molecular assays for MSI, IHC for dMMR, or EBV-encoded RNA in situ hybridization for EBV. Radiomics is most appropriately used within integrated models combining imaging, pathology, molecular biomarkers, and clinical variables rather than as a stand-alone surrogate for tumor immunity.

## 3 Applications of deep learning-based pathomics in TME characterization and immunotherapy response prediction in GC

Unlike radiomics, pathomics directly captures cellular morphology and tissue architecture from WSIs, including immune-cell density, spatial distribution, and cellular neighborhood within tumor parenchyma and stroma (28, 76). However, lineage-specific immune phenotypes generally require immunohistochemical or molecular annotations to serve as ground-truth labels for training deep learning models, as these phenotypes are not directly visible on routine hematoxylin and eosin (H&E) images. Deep learning enables automated and high-throughput extraction of these histologic features associated with TME states, molecular biomarker expression, and immunotherapy outcomes, thereby facilitating spatially resolved analysis of tumor morphology and intratumoral heterogeneity across large tissue areas, while extending routine histology beyond visual assessment.

### 3.1 Deep learning-based pathomics for quantifying cellular and spatial TME organization

Although conventional IHC remains the reference method for lineage-specific immune-cell identification, deep learning-based pathomics extends the scope of conventional cell counting by quantifying cellular composition and inferring TME phenotypes from histological images. Chen et al. (77) demonstrated that immune-inflamed, immune-excluded, and immune-desert subtypes exhibit distinct immune cell abundance patterns, and then trained a ResNet-18 model on H&E-stained WSI patches to capture their corresponding histomorphological correlates, achieving accuracies of 76.47%–85.71% across the training, validation, and test cohorts. In contrast, Wei et al. (78) quantified inflammatory, connective, and necrotic cells within tumor stroma and identified higher inflammatory-cell proportions in EBV-positive, MSI-H, and TMB-high GCs, linking H&E-derived cellular composition to molecular phenotypes associated with immunotherapy sensitivity. Collectively, H&E-derived cellular composition may provide a tissue-level correlate of established immunotherapy biomarkers, but its incremental predictive value beyond molecular testing remains unproven. Although H&E morphology cannot establish immune lineage as specifically as IHC, these measurements directly capture histological composition and spatial context that molecular assays cannot provide.

Beyond cellular abundance, tertiary lymphoid structures (TLSs) are organized lymphoid aggregates consisting of B cells, T cells, dendritic cells, high endothelial venules in mature structures, and other immune components, thereby reflecting organized local antigen presentation, lymphocyte recruitment, and adaptive immune responses within the TME (79). Recently, TLSs have been associated with improved immune activation and favorable responses to immunotherapy in several cancer types (80, 81). Chen et al. (82) trained a deep learning network to automatically recognize and segment TLSs and calculate the TLS ratio, defined as TLS area divided by tissue area. The associations of the TLS ratio with B-cell abundance and *CXCL13* expression supported construct validity but did not establish TLS maturity, cellular composition, or antigen-presentation capacity. Overall, pathomics studies should distinguish direct morphometric quantification from surrogate inference of molecular or functional TME states.

As research advances, WSI-based characterization of the TME may be extended to infer selected gene mutations and pathway-associated activity signatures (83). This emerging approach to comprehensive TME quantification is potentially cost-effective and reproducible, and it may complement existing biomarkers, thereby enabling more accurate identification of patients with GC who are likely to benefit from immunotherapy.

### 3.2 Deep learning-based pathomics for predicting molecular biomarker status and gastric cancer subtypes

EBV-positive and MSI-H/dMMR GCs frequently show lymphocyte-rich, immune-inflamed histological features (23, 84). These tumor phenotypes can generate recognizable epithelial, stromal, and immune patterns in H&E sections (22), providing a morphological basis for inferring immunotherapy-related molecular biomarkers from routine H&E slides. Unlike models trained on clinical treatment outcomes, models trained on molecular biomarker labels predict molecular expression status rather than survival outcomes, and may prioritize cases for confirmatory testing when ancillary testing capacity is limited (23, 85, 86).

Kather et al. (87) provided an early proof of concept that deep learning could predict MSI status directly from H&E-stained sections in gastrointestinal cancers, establishing the feasibility of linking histomorphology to molecular alterations. Translating this concept into a GC-specific application, Hinata et al. (88) developed a VGG16-based CNN model to identify the combined EBV-positive and MSI/dMMR subgroup, achieving AUCs of 0.947 in the internal test cohort and 0.870 in an external cohort. Model visualization highlighted intraepithelial lymphocytosis in the heat map, suggesting that the classifier relied on a shared lymphocyte-rich morphology associated with these molecular subtypes rather than directly measuring sensitivity to ICIs. Wei et al. (78) developed deep learning models to infer EBV, MSI, TMB, and PD-L1 expression directly from H&E-stained WSIs, achieving internal AUCs of 0.869–0.946. However, the external validation was limited to EBV and PD-L1, with AUCs of 0.791 and 0.638, respectively. The decline in external performance suggests limited cross-cohort transportability and highlights the need for geographically and technically diverse cohorts, which enable characterization and mitigation of center-specific performance loss.

Consistent with this need, Muti et al. (89) evaluated separate MSI and EBV classifiers across ten cohorts from seven countries. The marked intercohort variation in performance, together with the improved robustness achieved through pooled multicohort training, demonstrated the feasibility of histology-based molecular inference and revealed the challenges of transporting such classifiers across heterogeneous clinical settings. Focusing specifically on EBV status, Zheng et al. (90) developed EBVNet and achieved AUCs of 0.941 and 0.895 in The Cancer Genome Atlas of Stomach Adenocarcinoma (TCGA-STAD) and MultiCenter-STAD cohorts, respectively, showing that a biomarker-specific model can retain strong discriminatory performance across external datasets.

Collectively, these studies demonstrate that H&E-stained images contain reproducible morphological correlates of selected molecular states. However, their external performance remains dependent on the biomarker, cohort composition, and technical workflow. Multicohort training can improve robustness but does not eliminate the performance gap caused by differences in slide preparation, scanner type, and patient population. Therefore, WSI-based classifiers are currently better suited to prescreening and prioritizing confirmatory testing than to replacing molecular assays for MSI, EBV-encoded RNA *in situ* hybridization, or IHC for dMMR.

### 3.3 Deep learning-based pathomics for predicting immunotherapy response in gastric cancer

As immunotherapy has become an established treatment option for GC, researchers have begun to explore the feasibility of using deep learning-based pathomics to assess immunotherapy benefit as a pretreatment tool. By capturing histologic information across multiple dimensions, including cellular morphology, TME architecture, and single-cell spatial organization, these models may support pretreatment prediction of response and stratification of PFS, although their clinical utility remains unproven.

In the STAD cohort analyzed by Chen et al. (82), a higher TLS ratio was associated with response to anti-PD-1 therapy, in which responders had higher pretreatment TLS ratios than nonresponders. Han et al. (91) used deep learning to extract nuclear features, microenvironmental features, and single-cell spatial-distribution features from pretreatment WSIs. Beyond response classification, the model-derived signature was independently associated with longer PFS after adjustment for clinicopathological variables. Pathogenomic analysis further linked model-predicted response to complement activation, interferon-γ response, and inflammatory-response pathways, as well as to higher immune scores and lower tumor purity, suggesting that the model captured an integrated morphology of an immune-active TME rather than a single histological feature. Nevertheless, these studies were retrospective and included only patients receiving ICIs. Without a non-ICI-treated comparator, these models cannot distinguish treatment-specific predictive effects from general prognostic associations.

As mentioned above, radiomics and pathomics provide complementary rather than redundant views of the TME. CT-based radiomics captures whole-tumor and peritumoral heterogeneity non-invasively but represents immune states only indirectly (29), whereas pathomics resolves cellular morphology and spatial tissue organization but is limited by tissue sampling and the lineage specificity of routine H&E staining. Radiomics may therefore be more suitable for longitudinal monitoring, while pathomics provides stronger tissue-level biological interpretation. This macroscopic-to-microscopic complementarity provides the rationale for multimodal integration (28, 77, 78, 91).

### 3.4 Comparison and complementarity between deep learning pathomics and established biomarkers

Although deep learning-based pathomics has shown promise for TME characterization, molecular biomarker inference, and ICI-response prediction, direct evidence of incremental value beyond established biomarkers remains limited in GC. Han et al. (91) directly compared a pathomics-driven ensemble model with PD-L1 CPS for ICI-response prediction. The model achieved AUCs of 0.921, 0.914, and 0.927 in the internal and two external validation cohorts, respectively, compared with 0.581, 0.459, 0.508 for CPS, with significant differences by DeLong’s test. These findings suggest that quantitative histomorphology may capture tumor, stromal, and immune spatial information not fully represented by PD-L1 expression alone.

By contrast, direct comparisons with MSI/MMR, EBV, or TMB for the same immunotherapy-response endpoint remain scarce. Muti et al. (89) reported external-validation AUCs ranging from 0.723 to 0.863 for MSI classification and 0.672 to 0.859 for EBV classification across independent cohorts. Consistently, a meta-analysis of 12 GC studies reported a synthesized concordance index (C-index) of 0.86 (95% CI: 0.83–0.89) for MSI prediction using pathology image-based models (92). However, both the individual and pooled estimates reflect molecular-status classification rather than ICI response. Therefore, high AUCs for molecular-status classification should not be interpreted as evidence that pathomics provides incremental predictive value beyond tissue-based biomarkers. Future studies should directly compare pathomics, established biomarkers, and their combination within the same ICI-treated cohorts, with evaluation of discrimination, calibration, decision-curve net benefit, and treatment-specific outcomes (93–96). A formal pooled AUC comparison is currently constrained by head-to-head studies and substantial heterogeneity in endpoints, treatment regimens, and cohort composition (97, 98).

## 4 Deep learning-driven multimodal fusion for immunotherapy biomarker development

The integration of radiomics, pathomics, and clinical data may improve the predictive performance and clinical relevance of deep learning models in GC immunotherapy by fusing macroscopic imaging phenotypes with microscopic immune contexture. The cross-scale complementarity between radiomics and pathomics, and the resulting multimodal approaches, are summarized in **Table 1**.

**Table 1:** Cross-scale complementarity of radiomics, pathomics, and multimodal approaches for decoding the gastric cancer immune microenvironment.

Approach	Data source	Spatial/biological scale	TME information	Advantages	Main limitations
Deep learning radiomics	Baseline CT/MRI	Whole-tumor, peritumoral regions, and metastatic lesions; macroscopic scale	Immune stromal, vascular, and necrotic phenotypes captured indirectly	Non-invasive and reproducible whole-tumor assessment	Cannot directly resolve cell lineages or signaling pathways
Delta radiomics	Pre- and post-treatment CT	Temporal dimension combined with whole-tumor assessment	Early treatment-related phenotypic changes	Enables monitoring the changes in both TME and tumor	Highly sensitive to scan segmentation methods
Deep learning pathomics	H&E-stained WSI	Local tissue, cellular, and cell-neighborhood scales	Cellular morphology, spatial organization of immune cells, stromal features, and TLSs	High histopathologic interpretability	Susceptible to biopsy sampling bias, cannot distinguish all immune cell lineages
Radiopathomics	CT, H&E-stained images, and clinical/molecular biomarkers	Cross-scale integration from macroscopic to microscopic levels	Integration of whole-tumor heterogeneity with the local immune ecosystem	Complementary information across modalities	Missing modalities, overfitting, and complexities in image registration

CT, computed tomography; H&E, hematoxylin and eosin; MRI, magnetic resonance imaging; TME, tumor microenvironment; TLSs, tertiary lymphoid structures; WSI, whole-slide image.

### 4.1 Integration of radiomics with clinicopathological variables

Among these, the most common approach combines radiomic signatures with clinicopathological variables, including tumor location, size, differentiation, Lauren classification, serum tumor markers, and TNM stage (99). Although clinically accessible, these variables largely reflect tumor phenotype and disease burden and may not capture the TME, intratumoral heterogeneity, or peritumoral microenvironment features, limiting their performance in heterogeneous GC (100).

In this context, radiomics has emerged as a promising noninvasive tool to complement traditional clinicopathological parameters. Sun et al. (101) developed two CT-derived imaging biomarkers, lymphoid radiomics score and myeloid radiomics score, which were more important than clinicopathological variables for classifying the TME immune context. Radiogenomics analyses further linked a high lymphoid radiomics score and low myeloid radiomics score to immune activation and tumor-suppressive signaling, including *p53*-mediated apoptosis and inflammatory responses, whereas the opposing phenotype was correlated with oncogenic pathways such as *MYC*- and *E2F*-driven proliferation and metabolic reprogramming. These findings suggest that CT-derived features may reflect distinct immune functional states within the TME. Whether such imaging surrogates of immune states translate into improved prognostic accuracy, however, requires direct comparison with established clinical indicators. Gou et al. (57) addressed this question by integrating the CT model with PD-L1 CPS expression and the lung immune prognostic index in a nomogram, which improved 1- and 2-year survival prediction. The integrated nomogram yielded C-indices of 0.85 and 0.78 in the training and external validation cohorts, respectively, and provided greater decision-curve net benefit than any single variable for 1-year survival in the training cohort. These findings suggest that imaging and systemic indicators provide complementary prognostic information for immunotherapy-treated populations.

### 4.2 Radiopathomic fusion across spatial scales

Beyond augmenting a single imaging modality with clinicopathological variables, radiopathomic fusion offers complementary tumor information across spatial scales. As mentioned above, pathomics resolves cellular morphology and organization but is constrained by tissue sampling, whereas radiomics surveys the entire tumor and surrounding tissues but lacks cellular resolution. This complementarity provides a rationale for combining the two modalities to achieve a more comprehensive representation of tumor heterogeneity. Wang et al. (102) integrated CT and H&E-derived features into a radiopathomics signature associated with immunotherapy response (hazard ratio: 3.651, *p* = 0.009) and effectively stratified patients by PFS. Moreover, Huang et al. (103) combined CT radiomic and deep learning features with H&E-derived microenvironmental, cell-density, and nuclear features across a multicenter cohort. Their signatures achieved AUCs of 0.978, 0.863, and 0.822 in the training, internal validation, and external validation cohorts, respectively, outperforming unimodal models and conventional biomarkers such as CPS and MSI-H. Thus, cross-scale fusion may narrow the information gap between macroscopic imaging and spatially restricted tissue samples.

### 4.3 Challenges and limitations in multimodal fusion

The design characteristics, biological reference standards, validation strategies, and major limitations of representative studies are summarized in **Table 2**. However, the clinical translation of multimodal fusion models remains constrained by several methodological and practical challenges. Complex multimodal deep learning models trained on limited cohorts are prone to overfitting (104). Although nested cross-validation can reduce optimistic bias during model selection (105), independent multicenter and prospective validation remains necessary to establish generalizability. While modality dropout, missing-modality-aware fusion, imputation, and cross-modal knowledge distillation may mitigate the challenge of incomplete multimodal data (106), external validation remains lacking for immunotherapy-specific cohorts. Moreover, the routine availability of CT and H&E slides does not ensure seamless implementation because segmentation, slide digitization, preprocessing, and quality control may still impose substantial workflow requirements. Finally, complex fusion architectures require clinically meaningful explanations and accessible visualization tools. Feature attribution maps alone are insufficient unless their robustness and biological relevance are validated (107).

**Table 2:** Representative deep learning-based radiomics, pathomics, and multimodal studies for tumor microenvironment characterization and immunotherapy-related outcome prediction in gastric cancer.

Study	Modality and architecture	Cohort design	Biological ground truth/training labels	Immunotherapy endpoint	Biological interpretation	Key limitations
Wang et al. (2)	CT; clinical variables	Non-ICI-treated used as unlabeled data, anti-PD-1 cohort	ICI responders and nonresponders according to RECIST	ORR in anti-PD-1 cohort	Imaging phenotypes complementary to CPS	Lacking an outcome-labeled non-ICI comparator and other biological and spatial validation.
Jiang et al. (54)	CT; biology-guided MTL deep learning	Non-ICI-cohort; ICI cohort	TME phenotypes based on IHC-derived IS/POSTN	ORR, PFS in ICI cohort	TME1: high IS/low POSTN; TME2: high IS/high POSTN; TME3: low IS/low POSTN; TME4: low IS/high POSTN	Lacking independent external ICI validation, retrospective design and possible selection bias
Sang et al. (55)	CT; clinical data; MTL based on AER-SwinT	Chemotherapy cohort; anti-PD-1 cohort	DFS-based chemotherapy-response; TME phenotypes based on IHC-derived IS/POSTN	ORR, PFS in ICI cohort	TME1: high IS/low POSTN; TME2: high IS/high POSTN; TME3: low IS/low POSTN; TME4: low IS/high POSTN	Primary task label derived from chemotherapy response
Gou et al. (57)	CT, clinical variables; Ensemble deep learning	ICI cohort	Binary 1-year OS: survival-positive (>12 months), survival-negative (<12 months).	OS only	A nomogram combining imaging, PD-L1 and LIPI	Lacking non-ICI cohort; no immunotherapy response assessment
Huang et al. (103)	CT, WSI-derived deep-learning features	Systemic ICI cohort, neoadjuvant immunotherapy cohort	ICI responders and nonresponders according to RECIST	PFS, OS in ICI cohort	Exploratory RNA-sequencing	Lacking non-ICI cohort; insufficient separation of chemotherapy and ICI effects
Wang et al. (102)	CT, WSI-derived deep-learning features	Surgical cohort; ICI cohort for exploratory validation	5-year OS in surgical cohort	ORR, PFS, OS	Exploratory ICI cohort validation	Small ICI cohort used only for exploratory evaluation
Han et al. (91)	WSI; Ensemble model	ICI cohort	ICI responders and nonresponders according to RECIST	ORR, PFS, OS	SHAP and TCGA pathogenomics analyses	Lacking non-ICI cohort; postoperative section slides

AER-SwinT, attention-enhanced residual Swin Transformer; CPS, combined positive score; CT, computed tomography; DFS, disease-free survival; ICI, immune checkpoint inhibitor; IHC, immunohistochemistry; IS, ImmunoScore; LIPI, lung immune prognostic index; MTL, multitask learning; ORR, objective response rate; OS, overall survival; PD-1, programmed cell death protein 1; PD-L1, programmed death-ligand 1; PFS, progression-free survival; POSTN, periostin; RECIST, Response Evaluation Criteria in Solid Tumors; SHAP, Shapley additive explanations; TCGA, The Cancer Genome Atlas; TME, tumor microenvironment; WSI, whole-slide image.

## 5 Clinical applications and translational challenges

### 5.1 Model interpretability and biological traceability for immunotherapy decision support

Although deep learning can capture complex TME-related signals from radiological and pathological images, high-dimensional abstract representations hinder biological traceability and clinical confidence (108). For immunotherapy decision support, predictive performance alone is insufficient. Models should also clarify which image regions and TME-related features contribute to individual predictions (54).

Biological traceability, referring to the ability to link computational representations with interpretable TME-related features, can be addressed at the architectural level. Two-stage architectures have been widely adopted to separate feature extraction from outcome prediction, enabling module-specific validation, feature selection, and model calibration. Similar strategies have been applied to survival and nutritional-risk prediction in GC (109, 110). This structural separation, however, does not guarantee that intermediate representations are independently accessible for biological validation.

Jiang et al. (111) leveraged this architecture to combine a deep learning radiomics signature with handcrafted CT features through a fully connected fusion module, and correlated the resulting signature with IHC-defined immune and stromal markers as well as TCGA-derived molecular pathways. Although this architecture comprises separable feature extraction and prediction stages, the entire network was trained end-to-end, meaning that the intermediate representations were neither extracted independently nor validated in isolation. Unless these intermediate features are explicitly exposed and evaluated, the immunologically relevant interpretability of even a nominally two-stage architecture remains limited.

By contrast, a hybrid model for MSI status prediction in GC used a two-stage architecture in which an intermediate deep learning-derived feature was explicitly extracted and could be independently evaluated for its predictive value (72). The deep learning feature alone achieved AUCs of 0.856 and 0.753 in the training and testing cohorts, respectively, whereas the final logistic regression model achieved AUCs of 0.883 and 0.802, indicating that the exposed representation retained predictive information and could complement conventional classifiers.

Beyond architectural transparency, post hoc methods provide complementary explanations. Shapley additive explanations (SHAP) analysis estimates the contribution of individual inputs to model predictions (101, 102), whereas attention maps highlight image regions associated with model attention patterns (107). For example, Sang et al. (55) used attention maps to visualize stage-dependent changes in spatial focus, where shallower layers attended to tumor boundaries and deeper layers increasingly emphasized intratumoral regions. These visualizations were made possible by the hierarchical, stage-wise architecture that exposes intermediate feature maps for post hoc interpretation.

At the clinical-presentation level, Gou et al. (57) constructed a nomogram integrating a CT-based deep learning ensemble score with clinicopathological variables to stratify patients by 1-year survival risk. In a three-center study of ICI-treated patients, Huang et al. (112) integrated a CT radiomics score with distant metastasis status to estimate 6-, 12-, and 24-month immunotherapy-related PFS. The resulting nomogram achieved C-indices of 0.687–0.778 across the training and validation cohorts. They further linked the radiomics score to M1 macrophage infiltration and inflammatory signaling, providing preliminary evidence of its molecular relevance to the TME.

Collectively, the interpretability of deep learning frameworks in GC immunotherapy remains heterogeneous across approaches. Architectural separation improves pipeline traceability but does not automatically validate the immune-related content of intermediate features. Post hoc attributions such as SHAP and attention maps reveal where models focus, but these observations only become biologically meaningful when linked to independent pathological or molecular evidence. Nomogram-based presentations increase clinical accessibility but likewise depend on the underlying validity of the imaging biomarkers they incorporate. Clinical interpretability therefore requires model attribution to be reproducible and concordant with pathological, molecular, and immunotherapy-treated cohort analyses, and these properties must be evaluated across multiple independent studies.

### 5.2 Model development and clinical translation in data-limited immunotherapy cohorts

Beyond interpretability, successful clinical deployment also requires robust model development strategies capable of overcoming limited checkpoint blockade cohort sizes. The relatively recent incorporation of ICIs into routine GC treatment has limited the size of available immunotherapy cohorts (113). Differences in treatment regimens, lines of therapy, combination strategies, and response assessment further reduce sample comparability. Although data augmentation and regularization may reduce algorithm variance, they cannot create independent biological or institutional diversity.

Semi-supervised learning can exploit routinely collected GC datasets without immunotherapy-response labels while reserving limited labeled cases for treatment-specific supervision. Rather than directly learning TME biology from unlabeled data, these algorithms learn disease-related image representations that may improve downstream response prediction. Wang et al. (2) trained a multimodal model on data from 168 patients with advanced GC receiving immunotherapy and incorporated data from 2,029 patients without immunotherapy-response labels. The framework achieved AUCs of 0.791 and 0.812 in the internal and external validation cohorts, respectively. This study illustrates how additional GC data can support representation learning when treatment-specific labels are scarce. The learned features still require validation in independently treated cohorts to determine their biological relevance.

Whereas semi-supervised learning exploits unlabeled cases within a related data domain, cross-task transfer reuses representations learned from a different clinical endpoint. Wang et al. (102) constructed a radiopathomics signature in a large non-immunotherapy cohort and then observed that higher signature values were associated with progressive disease and significantly shorter PFS than lower signature values in an immunotherapy cohort consisting of 64 GC patients. Similarly, Sang et al. (55) developed AER-SwinT in multicenter cohorts comprising 3,095 patients, using chemotherapy response as the primary task and two auxiliary TME-related tasks. When applied to two independent immunotherapy cohorts, the predicted TME classes were associated with anti-PD-1 response. These studies suggest that TME-related representations may retain cross-task value in data-limited immunotherapy settings. Because the immunotherapy cohorts were used mainly for evaluation rather than model adaptation, this approach is more accurately described as cross-task representation transfer than as conventional transfer learning.

### 5.3 Operational, regulatory, and clinical requirements for implementation

Beyond model explainability and biological traceability, clinical translation requires robust integration into routine workflows. Standardized imaging acquisition and preprocessing protocols are essential for reproducible model inputs. Meyer et al. (114) found that only 12 of 106 CT radiomic features remained reproducible across variations in radiation dose, slice thickness, and reconstruction settings, while harmonization can reduce scanner- and protocol-related variability (115, 116). Zhou et al. (117) further showed that models based on stable features maintained more consistent performance across CT settings (AUC, 0.693–0.728) than those based on unstable features (AUC, 0.523–0.800). Automated segmentation likewise requires quality control before downstream radiomic analysis. Specktor-Fadida et al. (118) developed a deep learning framework that estimated segmentation quality and localized potential errors. Pathomics additionally depends on validated slide digitization, scalable storage, and interoperability with hospital information systems. An international survey found that 55 of 127 pathology laboratories lacked a digital pathology workflow and only 29 used it diagnostically (119), whereas Schüffler et al. (120) demonstrated successful integration of WSI into four hospital systems, illustrating the feasibility of routine system-level deployment.

From a regulatory perspective, clinical deployment demands predefined procedures for model version control, data governance, post-deployment performance monitoring, and reassessment after substantial model updates or distribution shifts. These considerations are increasingly incorporated into regulatory oversight. The US Food and Drug Administration’s draft guidance for artificial intelligence-enabled device software functions adopts a total product life-cycle approach, including post-market performance monitoring (121).

Ethically, responsibility for deep learning-assisted decisions should remain clearly defined, with appropriate human oversight and safeguards for privacy and data-use (122, 123). Bias auditing is also essential, as Seyyed-Kalantari et al. (124) reported higher underdiagnosis rates across subgroups defined by sex, race and ethnicity despite overall model performance, while dataset imbalance, disease prevalence, and acquisition differences are recognized sources of unfairness in medical imaging artificial intelligence (125). Subgroup performance, calibration, and clinically relevant error rates should therefore be reported before routine implementation.

Finally, retrospective or external validation alone does not establish clinical utility. Jiang et al. (111) emphasized the need for prospective validation and randomized trials, while Han et al. (91) called for larger prospective cohorts despite external-validation AUCs above 0.90. Future studies should therefore use adequately powered multicenter prospective cohorts with standardized data acquisition and prespecified endpoints, followed by randomized evaluation when models are intended to influence treatment decisions.

## 6 Conclusions and future perspectives

Deep learning-based radiomics and pathomics interrogate the TME at different, non-interchangeable spatial scales. CT-based approaches sample the entire visible tumor and its peritumoral context, thereby capturing macroscopic heterogeneity that can be assessed repeatedly, whereas WSI approaches resolve cellular morphology, stromal architecture, and local immune organization within the sampled specimen (28, 29, 100, 111). This distinction explains both their respective value and their limitations. Radiological phenotypes cannot directly identify the cells or pathways that give rise to the observed imaging patterns, whereas a tissue section may characterize local immune ecology in detail but incompletely represent whole-tumor heterogeneity. Accordingly, the evidence synthesized in this review supports treating radiomics and pathomics as complementary views of the TME rather than competing substitutes for established biomarkers such as PD-L1 CPS, MSI/MMR, EBV, and TMB (15, 68–71, 91, 112).

Across these modalities, the most important advance is not simply improved discrimination, but the shift from isolated molecular surrogate markers toward representations connecting morphology, immune-stromal states, and treatment outcomes. Radiological models have associated CT phenotypes with immune contexture and response to checkpoint blockade, whereas pathological models have linked spatial and cellular patterns in routine H&E slides to immunotherapy response (54, 91, 100, 111, 112). Multimodal radiopathomics studies further suggest that combining whole-tumor imaging with tissue-level morphology can add information beyond either source alone. Such fusion is mechanistically informative when the two modalities capture related but non-redundant signals. Alignment through shared biological correlates, such as immune infiltration or stromal remodeling, may provide the basis for meaningful integration. However, immunotherapy analyses remain preliminary in some cohorts, without established treatment-specific utility (102, 103). Recent multicenter pathomics work continues to demonstrate the prognostic information encoded in H&E morphology, but reported immunotherapy sensitivity has often been evaluated indirectly or in limited treated cohorts, reinforcing the need to distinguish biological plausibility from validated prediction (126). Thus, the convergence of radiomics and pathomics is best interpreted as evidence for cross-scale complementarity, not as proof that adding more modalities will invariably improve clinical decisions.

The shared weaknesses of both evidence streams are more consequential than their algorithmic differences. Most studies remain retrospective, immunotherapy-treated cohorts are modest relative to model complexity, and populations differ in disease stage, treatment line, regimen, response definition, imaging acquisition, tissue processing, and scanner platform (88, 89, 104, 127–129). These sources of heterogeneity can produce domain shift, unstable calibration, and inflated performance that does not generalize to new centers. Multimodal models add a further selection problem because complete CT, digitized pathology, molecular testing, and longitudinal follow-up are not simultaneously available for every patient. Patient-level separation, center-held-out validation, locked-model testing, protocol harmonization, and explicit evaluation under realistic missing-modality patterns should therefore be regarded as minimum evidentiary requirements rather than optional refinements (94, 105, 115, 130, 131). Evaluation should report calibration, decision-curve net benefit, subgroup performance, and the clinical consequences of false-positive and false-negative classifications in addition to the AUC.

A further consideration across radiomics, pathomics, and multimodal studies is that associations with response or survival in ICI-treated cohorts are insufficient, by themselves, to establish treatment-specific predictive utility. Comparator cohorts and biomarker-by-treatment interaction analyses are needed to distinguish prediction of immunotherapy benefit from general prognostic stratification. The same principle applies across baseline radiomics, pathomics, and multimodal models, including models developed through semi-supervised or cross-task representation learning (2, 55, 102, 111, 112). Clinical use should be specified in advance. Pretreatment triage, complementary risk stratification, early response assessment, or detection of acquired resistance require different endpoints, sampling schedules, error tolerances, and reference standards. Until prospective utility is shown, models that infer MSI, EBV, PD-L1-related phenotypes, or other molecular states should be positioned as prescreening or test-prioritization tools rather than replacements for validated molecular, *in situ* hybridization, or immunohistochemical assays (72, 89–91).

Longitudinal imaging may complement single-time-point assessment by capturing treatment-related phenotypic changes that may reflect evolving TME states. In stage IV GC treated with ICIs, Li et al. (132) evaluated delta-radiomic changes between baseline CT and follow-up CT after two to three treatment cycles and identified an intratumoral texture change that was independently associated with PFS. Similarly, Wei et al. (133) used pre- and post-treatment CT in patients receiving PD-1 blockade plus neoadjuvant chemotherapy, with a delta-radiomics-based combined model achieving an external validation AUC of 0.913 for treatment response. Although current GC evidence has linked delta-radiomic changes to response or survival, whether these changes reflect dynamic TME remodeling has not been established. Complementary evidence from conventional radiomics provides biological plausibility: a CT radiomics-derived T-cell-inflamed signature was associated with intratumoral CD8, CD3, and CD163 expression and showed transitions between inflamed and uninflamed states across serial CT examinations during immunotherapy (134). Nevertheless, incremental value over Response Evaluation Criteria in Solid Tumors (RECIST) or standard biomarkers remains unclear (132), and a systematic review of 13 immunotherapy-related delta-radiomics studies found no prospective validation and generally limited external validation (135). Future GC studies should therefore prospectively standardize imaging time points and determine whether deep learning-derived longitudinal features track TME remodeling and acquired resistance by integrating serial imaging with matched pathological, molecular, or circulating immune measurements.

To improve the biological interpretation of longitudinal imaging changes, serial imaging should, where feasible, be paired with repeat tissue or circulating measurements that capture complementary aspects of the evolving TME. Circulating and exosomal PD-L1 measurements in GC illustrate this potential but have not constituted validated predictors of PD-1 blockade benefit (136–138). Likewise, attention maps, SHAP values, and feature attribution may identify image regions that influence a model, but they do not by themselves explain the underlying biology or provide reliable biological interpretation (39, 91, 107). Matched spatial transcriptomics, multiplex imaging, and genomic, transcriptomic, or proteomic data can test whether radiological and histological phenotypes correspond to immune infiltration, stromal activation, angiogenesis, or immune-evasion programs (54, 55, 139, 140). Causal or functional interpretation will still require reproducibility analyses and pathological or experimental validation.

Accordingly, explainable artificial intelligence (XAI) should be more systematically integrated into future radiomics and pathomics research to improve both model interpretability and clinical explainability. SHAP can provide both global and patient-specific estimates of feature contributions in radiomic, pathomic, and multimodal models, whereas complementary *post hoc* approaches, including gradient-based saliency maps, class-activation mapping, and attention visualization, can localize image regions driving model predictions. However, features identified by *post hoc* attribution methods should be further examined for biological relevance to determine whether their predictive importance corresponds to tumor biology or immune-related processes (141). Incorporating biological prior knowledge into feature construction and model development may facilitate this interpretation. For instance, an MRI study in rectal cancer derived lymph-node morphological and topological features based on transcriptomic and immunohistochemical evidence linking nodal characteristics to distinct immune states (142). Integrating biologically informed feature design with *post hoc* attribution and independent pathological or molecular validation may therefore provide a more robust framework for clinically interpretable predictions, thereby strengthening confidence in artificial intelligence-assisted clinical decision-making.

Personalized neoantigen mRNA vaccines can deliver tumor-specific antigens and induce neoantigen-directed T-cell responses, thereby enhancing antitumor immunity (143, 144). Beyond antigen delivery, mRNA platforms can encode immunostimulatory molecules, such as cytokines or costimulatory ligands, to modulate the TME. Clinical trials of intratumoral mRNA constructs encoding *OX40L*, *IL-23*, *IL-36γ*, or other immune-stimulatory cytokines have shown broad proinflammatory activity, increased T-cell infiltration, and changes in PD-L1 expression, providing a biological rationale for combining these platforms with immune checkpoint blockade (145). Although these clinical findings have mainly been obtained in advanced solid tumors rather than GC (146), a recent preclinical GC study showed that a lipid nanoparticle-formulated neoantigen mRNA vaccine induced CD8^+^ T-cell responses and enhanced antitumor activity when combined with anti-PD-1 therapy (147). These findings support further investigation of mRNA-based strategies as both antigen-delivery platforms and modulators of the TME in GC.

Taken together, current evidence supports deep learning-based radiomics and pathomics as complementary research biomarkers for decoding the gastric cancer TME, with encouraging but not definitive ability to predict immunotherapy outcomes (54, 91, 102, 103). The near-term translational priority should be task-aligned and modality-flexible development. Models should use the smallest set of routinely obtainable inputs that provides reproducible incremental value for a prespecified decision, while accommodating patients for whom tissue, serial imaging, or ancillary biomarkers are unavailable. Prospective multicenter studies must then demonstrate robustness, calibration, added value over PD-L1 CPS and MSI/MMR status, feasible cost and turnaround time, and an effect on treatment decisions or patient outcomes (94, 130, 131). If these conditions are met, radiomics may provide repeatable whole-tumor surveillance, pathomics may provide spatially resolved tissue context, and their integration anchored in shared biological correlates may support more precise patient stratification and response monitoring. At present, however, these methods should be viewed as adjuncts to multidisciplinary assessment and validated biomarkers, not as autonomous determinants of gastric cancer immunotherapy.
